# Principal component analysis on twenty years (2000–2020) of geochemical and geophysical observations at Campi Flegrei active caldera

**DOI:** 10.1038/s41598-023-45108-0

**Published:** 2023-10-27

**Authors:** Zaccaria Petrillo, Simona Tripaldi, Annarita Mangiacapra, Sergio Scippacercola, Stefano Caliro, Giovanni Chiodini

**Affiliations:** 1 Istituto Nazionale di Geofisica e Vulcanologia (INGV), Osservatorio Vesuviano, Via Diocleziano 328, Napoli, Italy; 2 Dipartimento di Scienze della Terra e Geoambientali, Università degli Studi di Bari Aldo Moro, via Orabona 4, Bari, Italy; 3 Istituto Nazionale di Geofisica e Vulcanologia, Sezione di Bologna, Via Donato Creti, 12, 40128 Bologna, Italy

**Keywords:** Geochemistry, Statistics

## Abstract

Campi Flegrei (CF) is an active and densely populated caldera in Southern Italy, which has manifested signs of significant unrest in the last 50 years. Due to the high volcanic risk, monitoring networks of the most sensitive unrest indicators have been implemented and improved over time. Precious database constituted by geophysical and geochemical data allowed the study of the caldera unrest phases. In this paper we retrace the caldera history in the time span 2000–2020 by analyzing displacement, seismicity and geochemical time series in a unified framework. To this end, Principal Component Analysis (PCA) was firstly applied only on geochemical data because of their compositional nature. The retrieved first three components were successively analyzed via PCA together with the geophysical and thermodynamical variables. Our results suggest that three independent processes relay on geochemical observations: a heating/pressurizing of the hydrothermal system, a process related to magmatic fluids injection at the hydrothermal system roots, and third process probably connected with a deeper magmatic dynamic. The actual volcano alert state seems mainly linked to the variation of the hydrothermal system activity. Our approach made it possible to explore the interrelation among observations of different nature highlighting the importance of the relative driving processes over time.

## Introduction

**Figure 1:**
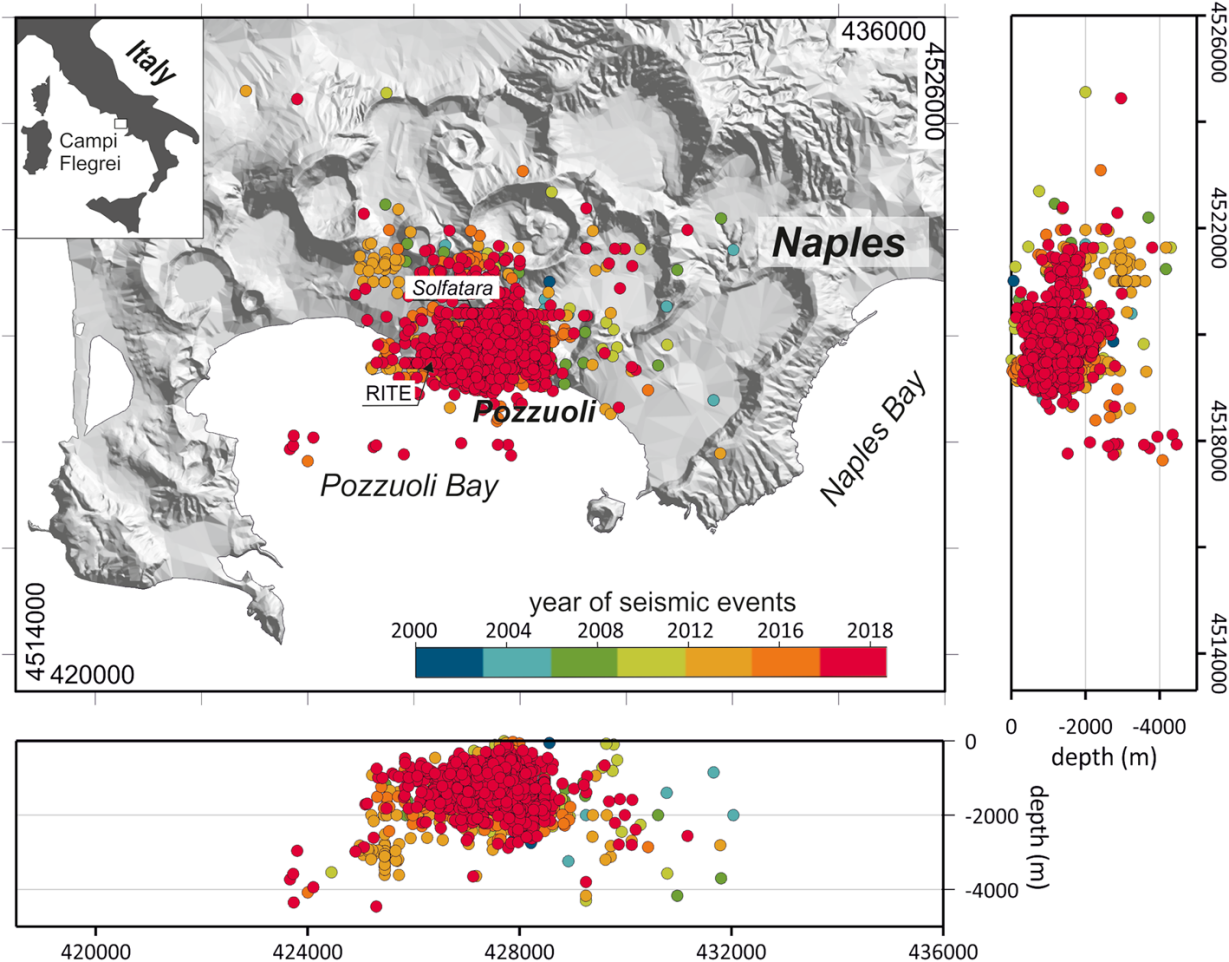
Campi Flegrei caldera map (UTM) showing the geochemical fluids sampling points (BG: zone 33T, Longitude 427649, Latitude 4519918), the ground lifting benchmark (RITE: zone 33T, Longitude 426319, Latitude 4519509), and earthquakes epicentres (1866 events with M ≥ − 0.5) in the period 2000–2020. On the right a depth-NS earthquakes projection and at the bottom the depth-EW earthquakes projection. Colours scale represents the earthquakes time occurrence. The software used to create the figure is Grapher 18.4.420 (https://www.goldensoftware.com/products/grapher).

In the last decades great attempts have been made to understand the complexity of the volcanic systems structures^1–3^ and to define the chemical and physical processes characterizing the associated hydrothermal systems^4,5^, united with their evolution through time. These attempts have been mainly addressed to the mitigation of the volcanic hazard in densely populated areas, where explosive catastrophic eruptions are expected. Campi Flegrei caldera (CFc, Fig. 1) in Southern Italy, represents a suitable example being a particularly dangerous active volcanic site, inhabited by more than 1.5 million people.

During its history, the caldera has experienced two very large explosive events that led to the formation of its primary structure: the Campanian Ignimbrite and the Neapolitan Yellow Tuff eruptions^6–8^ then modified by the more recent Agnano Monte Spina and Astroni explosive eruptions, as well as by the numerous successive eruptions, which generated pyroclastic deposits spread over an extremely large area^8,9^. The last eruption in CF occurred in 1538 AD^10^. Currently the caldera magmatic system is still active, as testified by bradyseismic episodes and by a widespread fumarolic and thermal springs^11^, leading this area to a very high volcanic risk.

Over the years the scientific community dealing with CF has mainly focused their attempt on improving the monitoring systems devices^12–20^ and developed numerous models to simulate volcanic and hydrothermal processes^4,21–29^. These efforts have been made in order to obtain reliable data and sophisticated simulators able to improve models confidence and predictive capabilities addressed to mitigate the volcanic risk and to prevent and estimate possible catastrophic scenarios.

Objectively, there is a certain difficulty to treat and interpret large sets of multiple variables data resulting from long-term monitoring. The basic strategy generally used is the univariate statistical analysis, which can cause, however, uncertainty and error when dealing with huge and inhomogeneous dataset^30^. In order to avoid this problem, multivariate statistical techniques can be used, as they are unbiased methods which can help indicate natural associations between samples and/or variables^31^, thus highlighting information not available at first glance.

In this study, we treat a large dataset of geophysical and geochemical data resulting from long-term (2000–2020) monitoring of the Campi Flegrei caldera. In particular, we examine, vertical displacements, earthquakes occurrences, geochemical composition of fumaroles and the estimated temperature and pressure time series using the Principal Component Analysis (PCA).

PCA is a multivariate statistical procedure widely applied for data processing and dimension reduction, when large multivariate datasets are analyzed. The PCA simplifies the data structure and helps data interpretation^32^. The great advantage^31^ lies in giving the ability to detect broader patterns of interrelationships among data than given by individual univariate analyses^30^. The main goal of this study is to identify and distinguish the different processes which in the last twenty years have been the protagonists of the ongoing crisis in the CF caldera and how these processes statistically interact. The geochemical fumaroles fluids samples, being compositional data, require a prior appropriate transformation according to theory of compositional data^33–38^. Via this transformation, it is possible to perform an analysis in Principal components to extract components which contain the synthesis of the geochemical processes acting in the CF caldera. Successively, the integration of the geochemical data with the geophysical data is carried out through a joint principal component analysis from which it is possible to identify the relationships between the physical processes and the geochemical variables synthesis involved in the analysis. The relationships found between geophysical and geochemical processes, allowed us to highlight the main process responsible for the last crisis.

## Materials and methods

### Compositional data analysis

All the statistical measures (e.g. mean, standard deviation, correlation, etc.) are defined in Euclidean space and, therefore, the usual univariate and multivariate analysis can lead to erroneous results when applied to compositional data. The constant sum for each observation limits the Euclidean sampling space to a Simplex, subspace of ${\mathfrak{R}}^{p}$. According to the current literature, only the transformation of compositional data by CoDA methodology^33–38^ allows a correct approach to the statistical data analysis. The three main approaches for modeling compositional data analysis are an *additive log-ratio* (*alr*), a *centered log-ratio* (*clr*)^34,39^ and the *isometric log-ratio* (*ilr*)^40,41^. The clr transformation is isometric^39^ and allows an interpretation of the relationships one-to-one among the compositional variables being a subspace of *p* dimensions^42^. Therefore, in this work, each compositional time series *X(t)* will be treated according to the *clr* methodology:

1 $${\varvec{Z}}\left( t \right) = clr\left[ {X\left( t \right)} \right] = \user2{ }\left[ {ln(x_{ij} \left( t \right)/g_{t} )} \right]\left( {i = 1, \ldots ,n;j = 1, \ldots ,p;t = t_{0} , \ldots ,T} \right)$$

where *n* is the number of observations*, p* the number of variables and* g*_*t*_ is the geometric mean of each compositional data at *t* time (*t ∈ T*).

### Principal component analysis and biplot

PCA linearly transforms the original variables into a smaller new set of uncorrelated variables^43^ each new variable is a linear combination of the old. The Principal components (PCs) are ordered so that the first few retain most of the information present in all of the original dataset. PCA can be implemented in two modalities: linear or via Decomposition into Singular Values (SVD)^44,45^*.* The first principal component is a linear combination of all variables with the greatest variability, the second principal component represents the greatest variability after the effect of the first has been removed, end so on. Since only the first components explain a significant part of the total variance, the remaining PCs can also be ignored. SVD is the most used method especially when one is interested in representing results with few dimensions. In this work we use SVD, following Aitchison's suggestion, since the compositional data have an adequate representation in the biplot. The Decomposition into Singular Values (SVD) of the ***X*** data matrix, centered or standardized is ${\varvec{X}}={\varvec{U}} {\varvec{\Lambda}} {\varvec{V}}\boldsymbol{^{\prime}}$, where ***U****(n,p)* are left eigen-vectors of ***XX’***, ${\varvec{\Lambda}}$*(p, p)* are the eigen-values, and ***V****’(p, p)* are the right eigen-vectors of ***X’X*** (***U*** and ***V*** are orthogonal). Splitting ${\varvec{\Lambda}}$ into ${{\varvec{\Lambda}}}^{\boldsymbol{\alpha }}{ {\varvec{\Lambda}}}^{\boldsymbol{\alpha }-1}$, where $\left(0\le \alpha \le 1\right)$. The SVD of matrix ***X*** becomes:

2 $$\user2{ } {\varvec{X}} = ({\varvec{U}} {\varvec{\varLambda}}^{{\varvec{\alpha}}} )\left( {{\varvec{\varLambda}}^{{1 - {\varvec{a}}}} \user2{V^{\prime}}} \right) = {\varvec{G}} {\varvec{H}}{^{\prime}}$$

where $${{\varvec{G}}={\varvec{U}} \boldsymbol{\Lambda }}^{\boldsymbol{\alpha }}$$ and $${\varvec{H}}={\boldsymbol{\Lambda }}^{1-\boldsymbol{\alpha }} {\varvec{V}}^{\prime}$$.

Biplot^34,46^ is a graphical tool that provides, in the factorial plane, the results of analysis in Principal Components (PCA) of a matrix ***X*** (*n, p*). The *bi* prefix indicates that the plot contains information on the *n* observations and on the *p* variables. In a biplot, the *n* rows of the matrix ***G*** (*n, 2*) are represented as points-units (scores), corresponding to the observations, and the *p* rows of the matrix ***H**** (p, 2****)*** are represented as vectors (rays), corresponding to the variables (loadings). The length of each vector starting from the origin of the axes, approximates the variance of the respective variable; the angle between two rays (cosine of the angles between the rays) approximates the correlation between the variables they represent; the projection of a generic data on a specific vector approximates the value of that data with respect to the variable represented by the vector. The *α* value in (2) can range from 0 to 1. In this work, we are interested to the covariance-biplot (α = 0) that preserves the covariance structure^47^ and privileges the display of the variables.

In the case of compositional data matrix, SVD can be applied after a clr, alr or ilr transformation^34^. After this application, the approximation structure of the correlations among data can be deduced. The biplot of compositional data (relative variance biplot), shows the rays (projection of the original variables onto this orthogonal space) and the links between two rays (the ratios among geochemical species). The links, that is the approximation of the geochemical ratio by the new subspace, are compositionally invariant, this mean they are good physical observables. If two links are orthogonal, an independent relationship between the two sub-compositions (equivalently the two sub-compositional ratios) is estimated; if the link between two rays is short or their vertices almost coincide, the relationship between the two log-ratios is constant and the two log-ratio are proportional^37,48^. The barycentre of Biplot represent the geometric mean, used in the clr transformation, and can be considered as a reference to evaluate the increase or the decrease of a variable respect to the whole composition. The ray length of the compositional data (for example the length of CO in Fig. 7) indicates the behaviour of the variable with respect to the centre of gravity (barycentre of Biplot) and provides an estimate of the standard deviation of a variable (approximately proportional to the own standard deviation, note that we are in a subspace, that is we have approximated the processes).

### Dataset and preliminary analysis

The raw geochemical dataset consists of time series of nine geochemical variables sampled with a frequency of about a sample/month; this dataset is constituted of 243 chemical analyses^49^ obtained from samples collected at the main fumarolic vent in Solfatara, Bocca Grande (BG, Fig. 1), between August 2000 and October 2020. Sampling techniques and analytical procedures are reported in Caliro et al.^12^. Chemical data of fumarolic fluids are expressed as micromole/mole (µmol/mol) for H_2_O, CO_2_, H_2_S, Ar, N_2_, CH_4_, H_2_, He, CO gas species. Fumarolic gases do not show any detectable SO_2_, HCl, and HF, due to the scrubbing of magmatic gases within the hydrothermal system^12,50,51^. The gas chemical compositions exhibit significant changes over time due to the periodic contributions of hotter and more oxidizing magmatic fluids entering the hydrothermal system^4,17,28,49,52–54^.

The examination of the behaviour of a single geochemical time series is distortive because the data are of a compositional nature^33^. Each statistical analysis is correct considering the ratios between two gas species (ratio is compositional invariant) although the number of independent ratios *[(p-1)*^*2*^*/2]* makes complex a global analysis of the geochemical data based on the ratios. We will use the PCA analysis to explore the structure of the compositional dataset and to obtain a new and reduced number of variables for further analysis.

Before applying the PCA, we used the ilr transformation to cut-out outliers and to resample geochemical data at a constant step of a sample/month. Then we back transformed data to the simplex space. In fact, the ilr subspace, having orthogonal axes and *p-1* dimensions, allows the interpolations of each variable independently from the others^33,55,56^. After the interpolation, the clr transformation was adopted.

**Figure 2:**
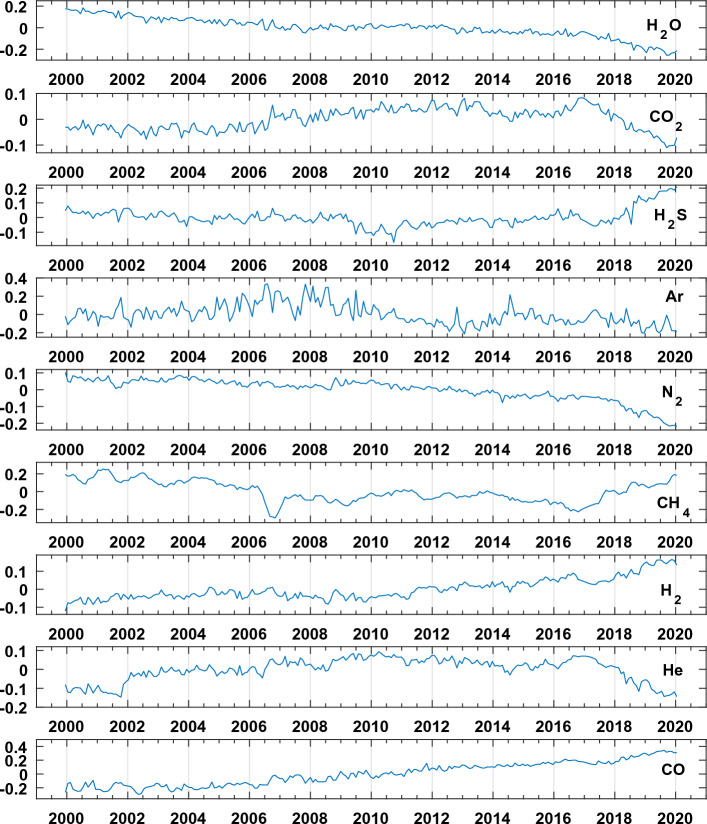
Gas species after centered log ratio (clr) transformation.

The geochemical time series resampled and transformed in *clr* are shown in Fig. 2, while their values are in the “Supplemental material”. In particular, it seems that, from a geochemical point of view, the crisis starts around the year 2006 with a clear fall of the CH_4_ content in the volcanic gasses with consequent increase in the typical ratios used to monitor the volcanic deep activity (CO_2_/CH_4_ and He/CH_4_).

Moreover, geochemical variables derived from gas equilibria have also been considered in the multivariate approach. Equilibrium temperatures and pressures, in the CO-CO_2_ gas system, are computed according to Chiodini^49^ considering the water fugacity controlled by the steam-liquid coexistence^57^ and redox conditions fixed by the D’Amore and Panichi^58^ empirical buffer.

**Figure 3:**
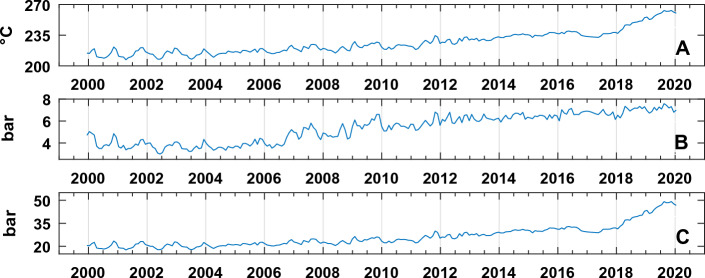
Estimated T–P from gas equilibria in the CO–CO_2_ system: (**A**) Temperature (in °C); (**B**) CO_2_ pressure (in bar); (**C**) H_2_O pressure (in bar) (see text for details).

Temperature is a function of ratio CO/CO_2_; H_2_O pressure is a function of Temperature; CO_2_ pressure is a function of ratio H_2_/CO (all the derived quantities depend on compositional invariant variable); the derivation of the geothermometric and geobarometric function was computed according to Chiodini et al.^49^, considering (i) *f*_H2O_ fixed by the vapour-liquid coexistence and (ii* f*_O2_ as a function of the temperature. Redox conditions of Solfatara gases were assumed to be controlled by the DP buffer (log *f*_O2_ = 8.20—23,643/T). The correspondent geothermometric relations are: T = 3133.5 / (0.933- Log (X_CO_ /X_CO2_)). The geobarometric functions are: Log P_H2O_ = 5.510—2048/T, where the water pressure is assumed equal to water fugacity of saturated vapor (i.e. vapor–liquid coexistence for pure water^57^), Log P_CO2_ = 3.025 + 201/T -Log (X_H2_ /X_CO_) and P_tot_ ~ P_CO2_ + P_H2O_ (Fig. 3).

**Figure 4:**
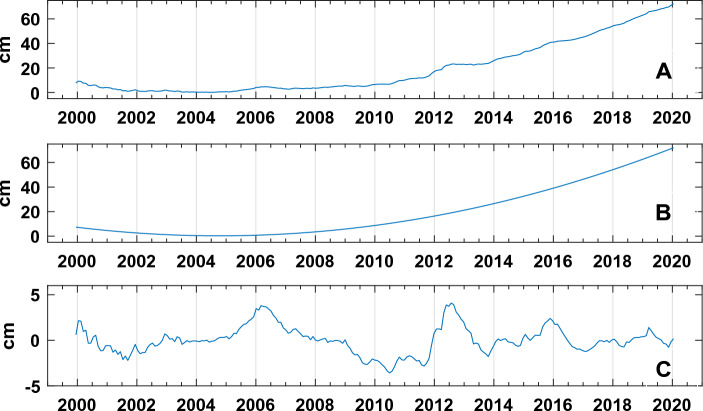
(**A**) Ground displacement in cm; (**B**) First geophysical variable (z-trend): Ground Displacement trend; (**C**) Second geophysical variable (z-osc): Ground displacement subtracted for the z-trend.

The vertical displacements dataset is composed of monthly averaged measurements recorded at the RITE station by the Neapolitan Volcanoes Continuous GPS (NeVoCGPS) network from 2000 to 2020 (Fig. 4A). The daily original recordings are available in Tramelli et. al.^59^. During the analyzed period, a subsidence phase switched to a slow uplift around 2005 and rose to a fast uplift phase in 2012 that is still ongoing. The maximum value was reached in the last analyzed year (2020) and was of 68.62 cm. Following the hypothesis that the complex displacement pattern is generated by the superposition of deformation processes separated in frequency (first intuited by Chiodini et al.^5^ and then formalized in Petrillo et al.^60^), the time series has been separated into two distinct time series: the trend obtained by a second order polynomial fitting (Fig. 4B) and the oscillating component (Fig. 4C) obtained by subtracting the trend. Hereafter, these two time series will be mentioned respectively as z-trend and z-osc. It is noteworthy that in the z-osc series is evident a peak around the year 2006 (as in the CH_4_ geochemical data series) as well as in the original up-lift time series where, around the same year, there is a step followed by an increasing trend (Fig. 4 A).

**Figure 5:**
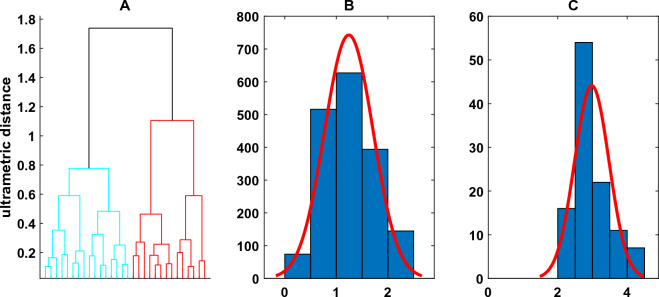
Earthquakes occurrences (1866 events) between 2000 and 2020: (**A**) Dendrogram of Earthquakes classified by depth: the left descending branch relates to Earthquakes with a hypocentral depth ≤ 2.36 km, the right descending branch relates to the deeper Earthquakes (hypocentral depth > 2.36 km); (**B**) Histogram of 1756 Earthquakes with hypocentral depths ≤ 2.36 km; (**C**) Histogram of 110 Earthquakes with hypocentral depths > 2.36 km.

The seismicity dataset is composed by monthly number of earthquakes located in the CFc area (Fig. 1). As reported by the catalogue of Osservatorio Vesuviano, Istituto Nazionale di Geofisica e Vulcanologia, between August 2000 up to October 2020, 2459 seismic events mainly occurred beneath the Solfatara-Pozzuoli area at depths from 0 to 4.46 km with the exception of a single (low quality and here not considered for further analysis) event at a depth of 7 km. The Gutenberg–Richter distribution^61^ closely fit the data with magnitude M ≥ − 0.5. In this study we have selected 1866 volcano tectonic earthquakes with M ≥ -0.5 at which 93% of the observed data (events) are modelled by a straight line^62^. From 2000 to 2020, seismicity increased in time and clustered to shallow depth. Analyzing the recent seismic activity, Chiodini et al.^54^ found that seismicity is distributed into low (swarms) and high (background) interarrival time populations^63–65^. The skewness of the hypocentral frequency distribution (in km) is 1.45, the mean is 1.34 with a standard deviation of 0.63, and the median is 1.27, which indicates a crowding of hypocentres towards shallow levels. We assume that there is a spatial discrimination between the two groups of earthquakes based on their depth. To test this hypothesis, a clustering algorithm (hierarchical linkage and Euclidian distance)^66^ on the hypocentral depths was applied. The resultant dendrogram (cophenetic index = 0.79) is shown in Fig. 5A.

**Figure 6:**
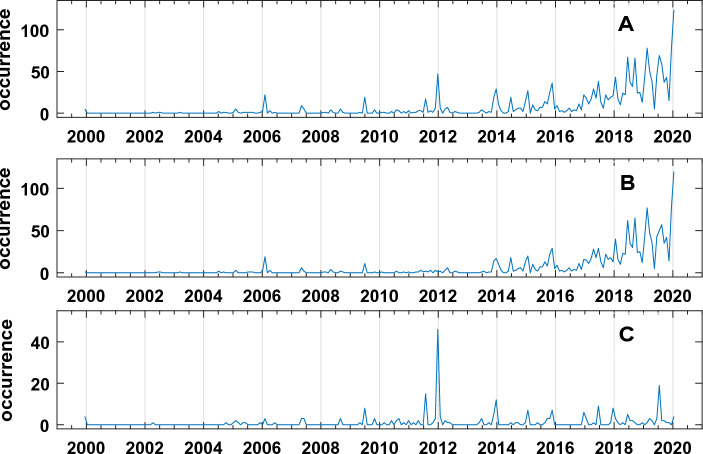
(**A**) Earthquakes occurrences; (**B**) Shallow Earthquakes occurrences (hypocentral depth ≤ 2.36 km); (**C**) Deep Earthquakes occurrences (hypocentral depth > 2.36 km).

Two distinct depth classes are evidenced (Fig. 5B,C): one between 0 and 2.36 km (first cluster, Fig. 5B) and the other below 2.36 km (second cluster, Fig. 5C). In Fig. 6 the two earthquakes time series related to the two clusters are shown. The very interesting result is the low value of temporal correlation (0.20) between the shallow and deep earthquakes time series, which, with the spatial clustering, strongly supports our hypothesis that there is the presence of two distinct (time/space) physical seismic processes. The structure of the earthquakes occurrence again shows, in a coherent manner with the others geophysical and geochemical variables, a significative peak of the earthquakes occurrence around the 2006.

In particular, the correlation between the original ground displacement (Fig. 4A) and the earthquakes time series related to the first cluster is 0.72, while the correlation between the ground displacement and the earthquakes time series related to the first cluster is 0.21.

Hereafter the shallow and deep earthquakes occurrence time series will be mentioned respectively as: s-E and d-E.

## Results and discussion

### Principal component analysis on geochemical species

**Figure 7:**
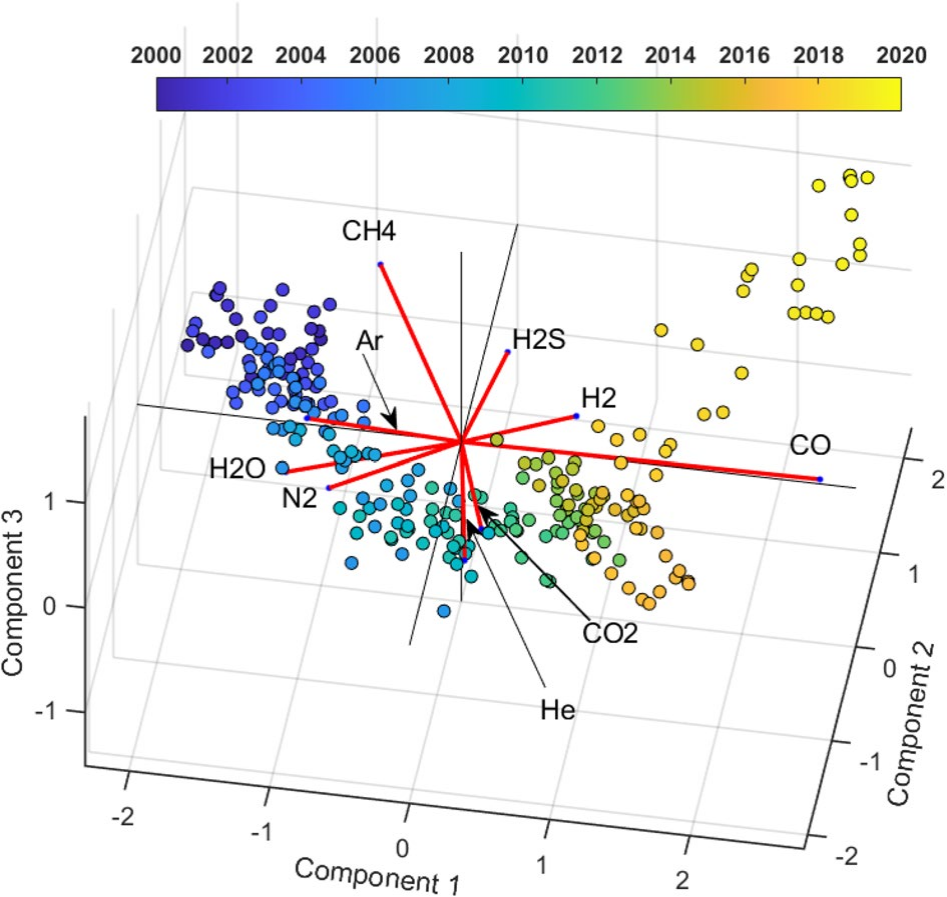
Relative covariance biplot of Centered Log Ratio (clr) Gas Species (in red) on the first three factorial planes (see text for details). Note that the scores distribution (colored dots), representing the system state, is not chaotic, it draws an evolutionary path.

To investigate the distribution of the geochemical variable in the reference periods, a PCA was applied on monthly sampled data, clr transformed by the Eq. (1) to obtain a relative covariance Biplot (Fig. 7) (Tables 1 and 2).

**Table 1:** Correlations among variables and PCs of the nine geochemical species in Fig. 7.

PC	H_2_O	CO_2_	H_2_S	Ar	N_2_	CH_4_	H_2_	He	CO
PC1 (GF1)	− 0.89	0.31	0.25	− 0.53	− 0.89	− 0.46	0.87	0.15	0.99
PC2 (GF2)	0.02	− 0.58	0.62	− 0.60	− 0.24	0.86	0.27	− 0.74	0.00
PC3 (GF3)	− 0.35	− 0.67	0.43	0.59	− 0.35	0.09	0.16	− 0.51	0.01

**Table 2:** Squared cosines referring to the PCA of the nine geochemical species in Fig. 7.

PC	H_2_O	CO_2_	H_2_S	Ar	N_2_	CH_4_	H_2_	He	CO
PC1 (GF1)	0.80	0.10	0.07	0.28	0.79	0.22	0.76	0.02	0.99
PC2 (GF2)	0.00	0.34	0.38	0.37	0.06	0.73	0.08	0.55	0.00
PC3 (GF3)	0.12	0.45	0.18	0.35	0.12	0.00	0.03	0.26	0.00

We look for hidden variables that control the changes of the fumarolic fluids compositions over time. To do this we will find the links which are best represented by the first three PCs (Tables 3, 4, 5).

**Table 3:** Correlations between the geochemical ratios and the first PC.

	H_2_O	CO_2_	H_2_S	Ar	N_2_	CH_4_	H_2_	He	CO
Numerator									
H_2_O		− 0.93	− 0.79	− 0.17	− 0.53	− 0.22	− 0.92	− 0.80	− 0.99
CO_2_	0.93		− 0.02	0.57	0.94	0.45	− 0.50	0.11	− 0.96
H_2_S	0.79	0.02		0.52	0.68	0.62	− 0.60	0.05	− 0.92
Ar	0.17	− 0.57	− 0.52		− 0.01	− 0.03	− 0.72	− 0.55	− 0.92
N_2_	0.53	− 0.94	− 0.68	0.01		− 0.03	− 0.91	− 0.86	− 0.99
CH_4_	0.22	− 0.45	− 0.62	0.03	0.03		− 0.75	− 0.39	− 0.91
H_2_	0.92	0.50	0.60	0.72	0.91	0.75		0.47	− 0.95
He	0.80	− 0.11	− 0.05	0.55	0.86	0.39	− 0.47		− 0.92
CO	0.99	0.96	0.92	0.92	0.99	0.91	0.95	0.92

**Table 4:** Correlations between the geochemical ratios and the second PC.

	H_2_O	CO_2_	H_2_S	Ar	N_2_	CH_4_	H_2_	He	CO
Numerator									
H_2_O		0.26	− 0.28	0.56	0.41	− 0.83	− 0.10	0.41	0.01
CO_2_	− 0.26		− 0.68	0.34	− 0.12	− 0.83	− 0.56	0.52	− 0.16
H_2_S	0.28	0.68		0.74	0.48	− 0.57	0.35	0.74	0.22
Ar	− 0.56	− 0.34	− 0.74		− 0.50	− 0.94	− 0.55	− 0.19	− 0.28
N_2_	− 0.41	0.12	− 0.48	0.50		− 0.94	− 0.27	0.37	− 0.07
CH_4_	0.83	0.83	0.57	0.94	0.94		0.61	0.88	0.41
H_2_	0.10	0.56	− 0.35	0.55	0.27	− 0.61		0.69	0.13
He	− 0.41	− 0.52	− 0.74	0.19	− 0.37	− 0.88	− 0.69		− 0.26
CO	− 0.01	0.16	− 0.22	0.28	0.07	− 0.41	− 0.13	0.26

**Table 5:** Correlations between the geochemical ratios and the third PC.

	H_2_O	CO_2_	H_2_S	Ar	N_2_	CH_4_	H_2_	He	CO
Numerator									
H_2_O		− 0.03	− 0.47	− 0.78	− 0.22	− 0.36	− 0.29	− 0.01	− 0.14
CO_2_	0.03		− 0.60	− 0.74	− 0.08	− 0.26	− 0.52	0.06	− 0.20
H_2_S	0.47	0.60		− 0.29	0.44	0.13	0.27	0.51	0.14
Ar	0.78	0.74	0.29		0.85	0.31	0.38	0.78	0.26
N_2_	0.22	0.08	− 0.44	− 0.85		− 0.27	− 0.27	0.11	− 0.11
CH_4_	0.36	0.26	− 0.13	− 0.31	0.27		0.01	0.25	0.03
H_2_	0.29	0.52	− 0.27	− 0.38	0.27	− 0.01		0.46	0.06
He	0.01	− 0.06	− 0.51	− 0.78	− 0.11	− 0.25	− 0.46		− 0.20
CO	0.14	0.20	− 0.14	− 0.26	0.11	− 0.03	− 0.06	0.20	

Looking at the biplot of the first three components (Fig. 7) (95% explained variance), CO and CH_4_ have the longest rays and, therefore, exhibit a much greater variability, in the time interval examined, respect to all the other geochemical species.

On the left side of the biplot (Fig. 7) there are the scores related to the period 2000–2005 (blue points) which reveal higher content in H_2_O, N_2_ and CH_4_. If we follow the scores evolution trough time, we can see that the system at t = 2000 is richer in CH_4_ than at t > 2005, when He and CO_2_ enter the hydrothermal system and start to play a major role in the score distribution (projection). In the transition period (from 2005 to 2017) He and CO_2_ mark a change in the hydrothermal system state evolution. From the year 2017 to 2020 we notice a decrease in the CO_2_ and He content and an increase of CH_4_ in the hydrothermal system. CO, which is the ray with higher variance, dominate the general trend.

In the context of the compositional theory, the analysis of individual species, in the clr space, leads to general results, but in particular we will use, even, the principal ratios (links) which are adopted in volcanic environments that can be geochemically interpreted. Therefore, we consider the correlation between the main ratios and the first three PCs (Tables 3, 4, 5).

**Figure 8:**
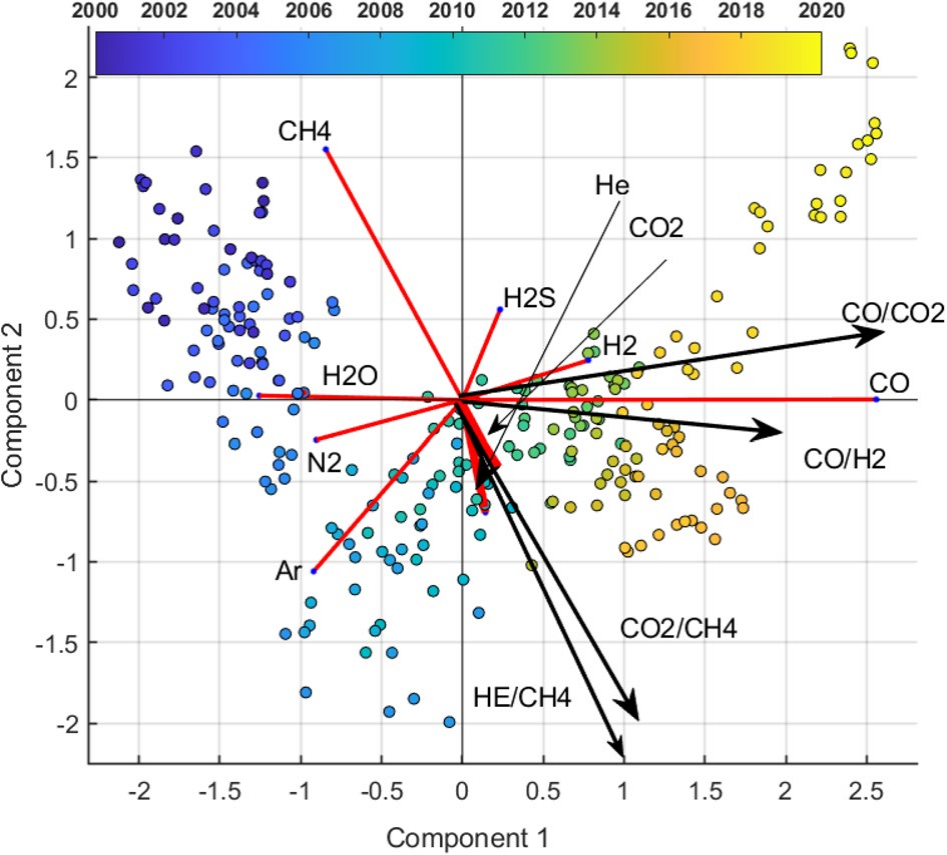
Relative covariance biplot of Centered Log Ratio (clr) Gas Species on the first factorial plane with the representation of main links (ratios) having geochemical significance.

The links (ratios) with geochemical significance which have high correlation with the first PC are CO/CO_2_ (r = 0.96) and CO/H_2_ (r = 0.95) (Table 3 and Fig. 8). This associations and these ratios are notable because CO and H_2_ are in general considered as gas species controlled by the Temperature and Pressure conditions of the hydrothermal systems at depth^67^. It should be emphasized that CO is related to all compositional species and all these relationships are strongly correlated with the first component (r ≥ 0.91). Considering that CO is the most representative species of the fumarole gas, delineating a warning trend^53^ of the hydrothermal system conditions, we can hypothesize that the first axis (Figs. 7 and 8) can be interpreted as a latent process of the hydrothermal system heating/pressurizing. In the follow, we name this first principal component as GF1 (first geochemical factor).

The second component orthogonal to the previous one, opposes CH_4_ and H_2_S to He, Ar and CO_2_ (Fig. 8 and Tables 1, 2). In the period 2000–2006 the second principal component is positively dominated by CH_4_ (which indicate a probably absence of deep magmatic fluids injection in the hydrothermal system) while, in the intermediate period 2007–2017, the contribution of He and CO_2_ determines a transition with a relative decrease of CH_4_. The last period (2017–2020) is again characterized by a relative increase of CH_4_. In the factorial plane, Ar is present in the transition period, however, its occurrence is not further discussed due to likely significant air contamination (see the wide high frequency oscillation in the Ar signal of Fig. 2).

Even for the interpretation of the second axis we resort to the analysis of the correlations between the geochemical ratios and the second component (Table 4). The ratios representative of the hydrothermal dynamics He/CH_4_ ratios (r = − 0.88) and CO_2_/CH_4_ (r = − 0.83), are well projected on the second component.

These last ratios have been suggested by Chiodini et al.^5,15,28,53,68^ as indicative of the arrivals of magmatic fluids in the hydrothermal system from depth. The CO_2_/CH_4_ and He/CH_4_ ratios have been interpreted as powerfull indicators of magma degassing episodes^65^. The magmatic gases entering the hydrothermal system are, in fact, relatively rich in CO_2_ and He and poor in CH_4_, a specie that is formed in the hydrothermal environment. Therefore, the second axis could represent a deep rooted hydrotermal process, that around 2006–2007 (see the projection of the data on the links CO_2_/CH_4_ and He/CH_4_ in Fig. 8) generated the pressure and temperature increase well represented by the first principal component.

We underline that the first component is very strongly correlated (angles among the rays and the first axis very near zero, Fig. 7) with the CO increasing trend (Fig. 2) and H_2_O reverse trend (Fig. 2). The second component, orthogonal to the first one, represents an independent process dominated by CH_4_. Note, orthogonality implies that, even if the second component (deep magmatic fluid batches) could be the cause of the first, their direct statistical dependence must have been lost over time. In the follow, we call the second principal component GF2 (second geochemical factor).

The third component (12% explained variance) is dominated during the intermediate period (2007–2012) by CO_2_ opposed to Ar and H_2_S (Fig. 7; Table 1). CO_2_ is the best represented among the geochemical species on this axis (Table 2). We can hypothesize that the third principal component (hereafter GF3, third geochemical factor) can be considered a latent factor linked to the production of CO_2_, probably from deeper zone of the volcanic system.

### Joint PCA on geochemical and geophysical data

**Figure 9:**
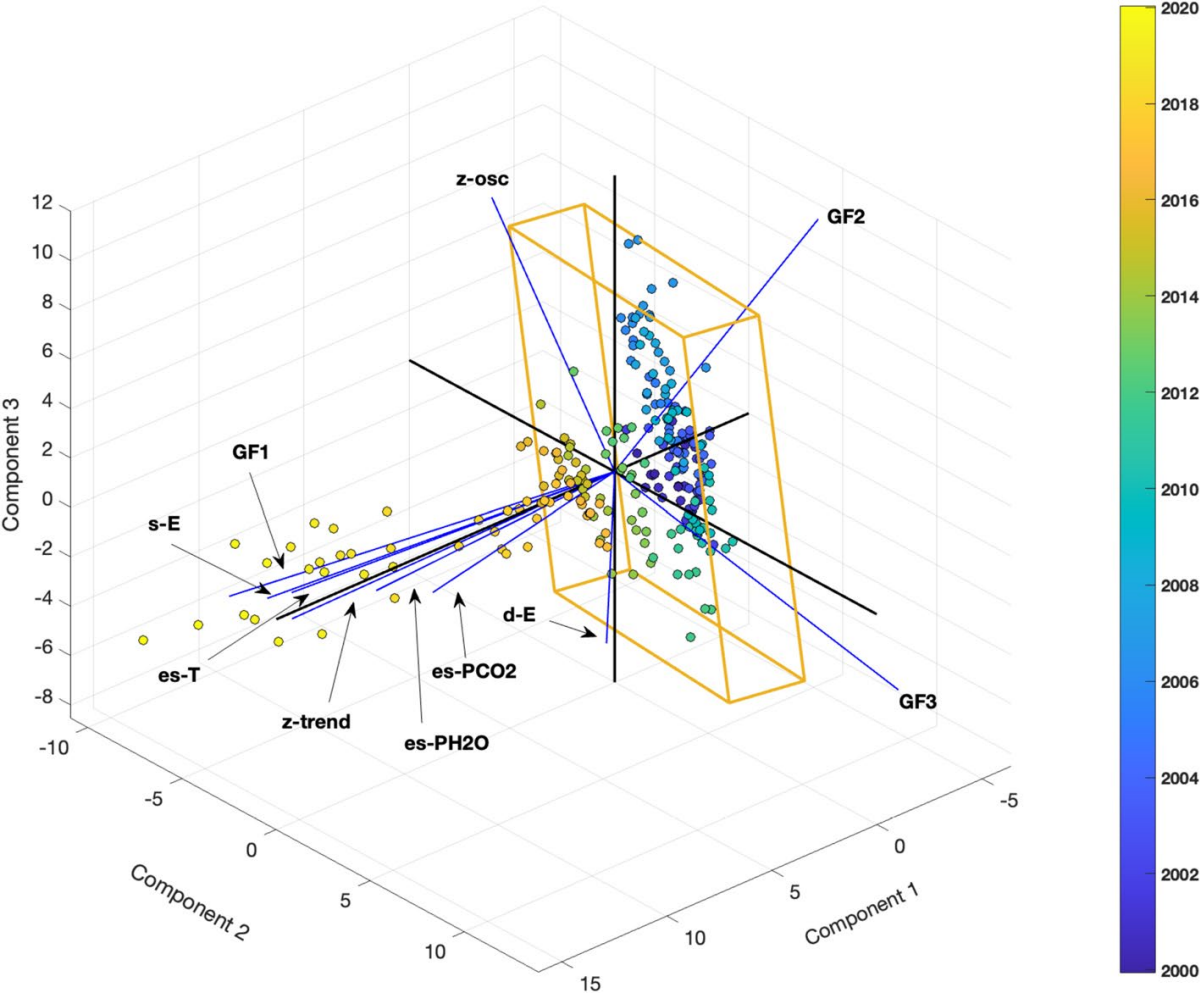
Biplot of the ground displacement trend (z-trend), ground displacement without trend (z-osc), shallow (s-E) and deep (d-E) Earthquakes occurrences, CO estimated Temperature (es-T), CO_2_ estimated pressure (es-PCO2), H_2_O estimated pressure (es-PH2O), GF1, GF2 and GF3. The explained variance is 80% in the first three factorial planes.

We were interested in the relationship over time among the geochemical data (Fig. 2), the geophysical data (Fig. 4B and C and Fig. 6B and C, and the derived T–P functions (Fig. 3). The Principal components analysis^69^ was conducted on the multivariate dataset where the geochemical data are represented by GF1, GF2 and GF3 obtained from the previous analysis. We found that the 85% of the total variance was explained by the first three components. The graphical representation of the Biplot in Fig. 9 (together with the inferences obtained from the data listed in Tables 6 and 7) shows an impressive development of the scores trajectory, representing the state of the system. The dynamic, in the early year (2000–2008), is constrained substantially in a volume (parallelepiped in Fig. 9) mainly described by the second and the third PC, here the dynamics is governed by GF2, GF3, deep earthquakes and z-oscillation. From the year 2009 up to 2014 the system is losing its stability and tends to invade the first PC. Around the year 2015 up to the year 2020, the system has a violent impulse and the scores pattern moves away from the parallelepiped along the first PC.

**Table 6:** Correlations among the first three PCs and the variables shown in Biplot of Fig. 9.

PC	z-trend	z- oscillation	Shallow earthquakes	Deep earthquakes	Est. Temp. CO	Est. Press CO_2_	Est. Press H_2_O	GF1	GF2	GF3
PC1	0.96	0.01	0.84	0.36	0.99	0.88	0.98	** 0.94**	− 0.21	− 0.19
PC2	− 0.03	− 0.40	− 0.38	0.37	0.00	0.36	− 0.08	0.24	0.47	** 0.75**
PC3	0.04	− 0.55	0.17	0.16	− 0.05	− 0.09	− 0.00	− 0.14	**− 0.79**	* 0.33*

Significant values are in [bold].

**Table 7:** Squared cosines of the variables referred to the Biplot in Fig. 9 (in bold the values that correspond. for each variable. to the factor for which the squared cosine is the largest).

PC	z-trend	z-oscillation	Shallow earthquakes	Deep earthquakes	Est. Temp. CO	Est. Press CO_2_	Est. Press H_2_O	GF1	GF2	GF3
PC1	**0.92**	0.00	**0.70**	0.13	**0.97**	**0.78**	**0.97**	**0.89**	0.04	0.04
PC2	0.00	0.16	0.14	**0.14**	0.00	0.13	0.00	0.06	0.22	**0.56**
PC3	0.00	**0.30**	0.02	0.02	0.00	0.03	0.00	0.02	**0.62**	0.10

Significant values are in [bold].

The first PC (Fig. 9) is strongly and positively correlated with the z-trend, CO estimated temperature, H_2_O estimated pressure and GF1. More moderate, but always very significant, is the association of shallow earthquakes and CO_2_ estimated pressure with the first component (Tables 6 and 7).

These correlations, together with the spatial correspondence between shallow earthquakes (located between 0 and 2.36 km at depth) and the hydrothermal system, support a strong link between the two. According to Chiodini et al., 2021 heating/pressurizing of the hydrothermal system plays an active role in triggering low magnitude seismicity at shallow depth^49^. The group of variables which characterize the first component implies a very interesting and generally original interpretation of the deformation trend which could be led by the hydrothermal heating/pressurizing in the first 2–3 km of the volcanic apparatus. This part of the hydrothermal system should be responsible of the deformation trend (bradyseism) as well as of the shallow earthquake occurrence. In fact, the advective/convective fluids transport mechanism increases the stress/strain by fluid pressure and so the earthquakes occurence. We interpret this first component, as representing fluids related processes occurring in the hydrothermal system.

The second component of PCA (Fig. 9) is strongly related only to GF3 (Tables 6 and 7) whose major variability is found around 2010–2012; note that the scores of this period are fully projected on the GF3 vector which, under the hypothesis that the GF3 factor is strongly correlated with the CO_2_, could be the trigger of the following crises, well represented by the first PCA (Fig. 9). This leads to interpret the second component (Fig. 9) as representing very deep volcanic processes related to the production of CO_2_. The second component has weaker negative correlations with z-osc and shallow earthquakes, while has weak positive correlations with GF2, deep earthquakes and CO_2_ estimated pressure. Notably this component shows an absolute independence from the z-trend.

**Figure 10:**
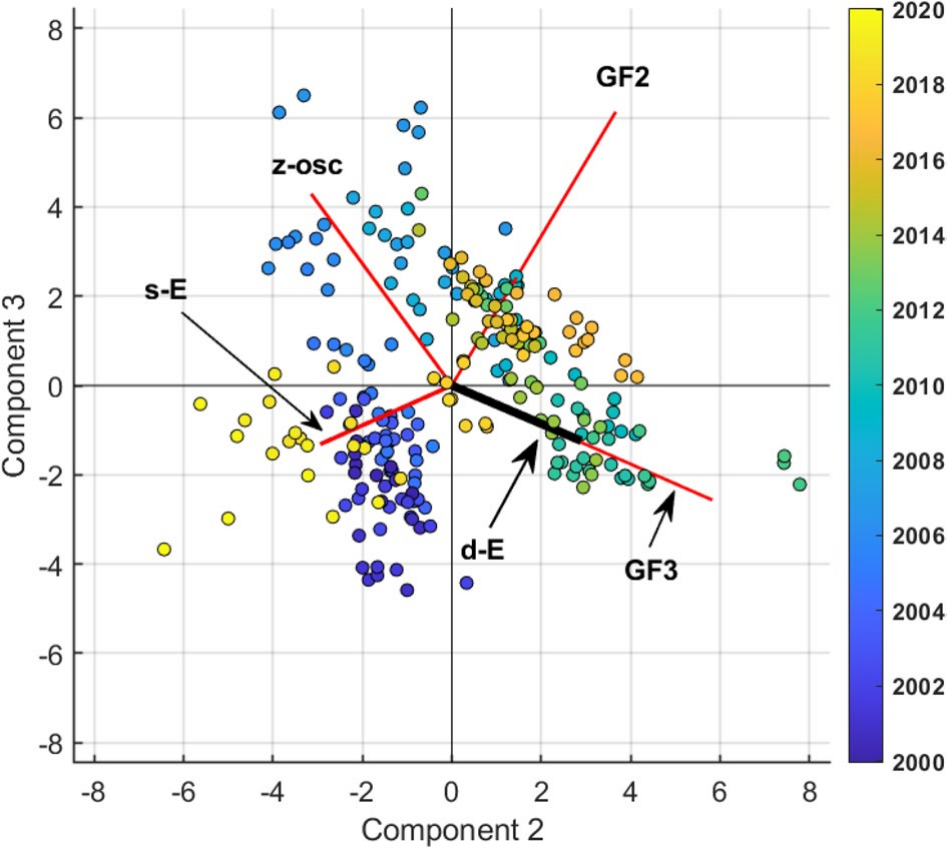
Biplot of only variables (in red and light blue) which have significant association with the second and third principal components (ground displacement without trend (z-osc), shallow (s-E) and deep (d-E) Earthquakes occurrences (the relative vector is dashed and in light blue), GF2 and GF3.

The third component of PCA (Fig. 9) is strongly related to GF2 (Tables 6 and 7) and to z-oscillations whose major variability is found around 2002–2012, from blue colour up to green in Fig. 10. Since GF2 could represents a process connected to the deep injection of magmatic fluids, we can suggest that the z-oscillations (in agreement with Chiodini et al.^5^) are generated by CH_4_—poor magmatic injections at the root of the hydrothermal system, probably below the region that hosts the most earthquakes (Fig. 1), as suggested by the very low correlations showed in Table 6.

## Conclusions

Twenty years of geochemical, thermodynamical and geophysical observations at CFc were analyzed by means of the PCA method. The goal was to reveal the basic phenomenon responsible for the ongoing state of a volcanic crises which started around 2006 and perturbed the physical–chemical CFc magmatic state. The current volcanic hazard level (yellow) has been determined by the increase in intensity and frequency of the main unrest indicators: earthquakes occurrence, ground deformation and volcanic gas flux at surface. The multivariate statistical analysis, we have applied, suggests processes occurring both in the hydrothermal and in the magmatic system, describing how these processes evolve through time.

The heating/pressurizing processes strongly dominate the multivariate space together with the deformation trend and the shallow earthquakes occurrences (first geochemical/geophysical PCA) during the whole period; however, the primary contribution to this PCA is driven by the recent steeply increase of these processes. We interpret this first component, as representing processes occurring in the hydrothermal system and dominating the ongoing unrest.

The second and the third components that consider modulation of the processes through the variables oscillation, show substantially a significant projection of GF2, GF3, deep earthquakes and z-oscillation. These components, associated to deep volcanic processes, dominate the years 2000–2012. Injection of deep heated fluids, at the base of the hydrothermal system, could be responsible for the deformations pulses, as already discussed in Chiodini et al., 2015^5^. The trigger of the CFc volcanic unrest could be driven by the GF3 variability.

In conclusion, we can state that the unified and integrated approach on geochemical and geophysical indicators, applied in this study, has allowed to reveal the hidden and independent processes at the base of the CF volcanic crises, not clearly identifiable considering just a subset of them.

The results of this study are the basis for the identification of further and perhaps more effective geochemical relationships useful to improve the monitoring of the evolutionary volcanic processes which affect calderas similar to the Campi Flegrei one.

The adopted strategy, using the compositional theory applied on geochemical data from the CF caldera, offers a global interpretative framework with the confirmation that the geochemical processes are a keystone in the interpretation of the volcanic phenomena at CF caldera. This methodology is certainly applicable to other calderas in the world in a similar state of hydrothermal activity.

### Supplementary Information

Supplementary Information.

## Data Availability

The datasets analyzed during the current study are available in the section: “Supplementary Information”.
